# Supplementary material for: Incidence and risk factors of calcium channel blocker–related edema in hypertensive patients: A Multicenter retrospective cohort study
Source: PLoS One. 2026 Mar 19;21(3):e0336659. doi: 10.1371/journal.pone.0336659 (PMC13001965; doi:10.1371/journal.pone.0336659)
Supplement: Attachment — Submitted filename: TL Perry comments Nov. 15, 2025 - PONE-D-25-58381_reviewer.pdf [file pone.0336659.s003.pdf]

PLOS ONE

Incidence and Risk Factors of Calcium Channel Blocker–Related Edema in Hypertensive Patients: A Multicenter Retrospective Cohort Study

--Manuscript Draft--

**Manuscript Number:**	PONE-D-25-58381
**Article Type:**	Research Article
**Full Title:**	Incidence and Risk Factors of Calcium Channel Blocker–Related Edema in Hypertensive Patients: A Multicenter Retrospective Cohort Study
**Short Title:**	Calcium Channel Blocker–Related Edema in HypertensiIon
**Corresponding Author:**	Gashaw Solela<br>Addis Ababa University College of Health Sciences<br>AA, ETHIOPIA
**Keywords:**	Adverse drug reactions; amlodipine; antihypertensive therapy; calcium channel blockers; Ethiopia; hypertension; peripheral edema.
**Abstract:**	Background: Hypertension is a major risk factor for cardiovascular disease and remains the leading cause of mortality worldwide. Calcium channel blockers (CCBs) are commonly used to lower blood pressure because they are effective and affordable. However, CCBs can cause vasodilatory adverse effects, including peripheral edema, which may lead to additional therapy and affect adherence. This study assessed the incidence and risk factors of CCB-related edema among hypertensive patients in Ethiopia.<br>Methods: This retrospective multicenter cohort study involved interviews and reviews of medical records of adults (aged ≥18 years) with essential hypertension who were prescribed calcium channel blockers (CCBs) between July 15 and August 14, 2025. A total of 292 participants were selected using systematic random sampling. A structured questionnaire was used to collect sociodemographic and clinical data. Descriptive statistics summarized baseline characteristics. Time-to-event analysis with the log-rank test assessed the duration from CCB initiation to edema onset. Binary and multivariate logistic regression analyses identified factors associated with CCB-related edema, and a p-value <0.05 was considered statistically significant.<br>Results: Among 292 participants (mean age 58.2 years; 53.4% female), 20.9% had diabetes mellitus and 16.8% had dyslipidemia. Amlodipine was the most frequently prescribed CCB (94.8%). Peripheral edema developed in 38.7% of patients, with a mean onset time of 8.3 weeks. In multivariate analysis, only longer daily standing duration was significantly associated with edema (AOR = 1.92; 95% confidence interval: 1.03–3.58; p = 0.041). Time-to-event analysis showed a progressive increase in edema risk with continued CCB use. Patients receiving amlodipine 10 mg daily had a greater (42.5% vs. 33%) and earlier risk of edema than those on 5 mg amlodipine daily; log-rank p = 0.003).<br>Conclusions: Peripheral edema occurred in over one-third of hypertensive patients on CCBs, appearing on average after eight weeks. Longer daily standing and higher CCB doses increased risk and earlier onset. Clinicians should inform patients and avoid unnecessary interventions. Further research on upright posture and edema is warranted.
**Order of Authors:**	Koricho Simie Tolla
	Gashaw Solela
	Getachew Wondafrash
	Abay Burusie
	Gebi Agero
	Dureti Desta Garoma
	Wubshet Abraham Alemu
	Bereket Sinshaw Engida

Powered by Editorial Manager® and ProduXion Manager® from Aries Systems Corporation

	Surafel Mekasha Woldeyes
	Berhanu Moges Abera
	Mulualem Alemayehu Gebreselassiea
**Additional Information:**	
**Question**	**Response**
**Financial Disclosure**<br><br>Enter a financial disclosure statement that describes the sources of funding for the work included in this submission. Review the submission guidelines for detailed requirements. View published research articles from *PLOS ONE* for specific examples.<br><br>This statement is required for submission and **will appear in the published article** if the submission is accepted. Please make sure it is accurate.<br><br>**Funded studies**<br>Enter a statement with the following details:<br>• Initials of the authors who received each award<br>• Grant numbers awarded to each author<br>• The full name of each funder<br>• URL of each funder website<br>• Did the sponsors or funders play any role in the study design, data collection and analysis, decision to publish, or preparation of the manuscript?<br><br>Did you receive funding for this work?	The author(s) received no specific funding for this work.
**Competing Interests**<br><br>Use the instructions below to enter a competing interest statement for this submission. On behalf of all authors, disclose any competing interests that could be perceived to bias this work—acknowledging all financial support and any other relevant financial or non-financial competing interests.<br><br>This statement is required for submission	The authors have declared that no competing interests exist.

and **will appear in the published article** if the submission is accepted. Please make sure it is accurate and that any funding sources listed in your Funding Information later in the submission form are also declared in your Financial Disclosure statement.<br><br>View published research articles from *PLOS ONE* for specific examples.<br><br>**NO authors have competing interests**<br><br>Enter: *The authors have declared that no competing interests exist.*<br><br>**Authors with competing interests**<br><br>Enter competing interest details beginning with this statement:<br><br>*I have read the journal's policy and the authors of this manuscript have the following competing interests: [insert competing interests here]*<br><br>* typeset	
**Ethics Statement**<br><br>Enter an ethics statement for this submission. This statement is required if the study involved:<br><br>• Human participants<br>• Human specimens or tissue<br>• Vertebrate animals or cephalopods<br>• Vertebrate embryos or tissues<br>• Field research<br><br>Write "N/A" if the submission does not require an ethics statement.<br><br>General guidance is provided below. Consult the submission guidelines for detailed instructions. **Make sure that all information entered here is included in the Methods section of the manuscript.**	Ethical clearance was obtained from the College of Health Science, Arsi University, Ethical Review Committee (Ref.No: A/U/H/S/C/120/13619/17). Written informed consent was obtained from all participants. Data were anonymized to protect confidentiality, and only relevant research information was collected. Clinical care and research activities were strictly separated, with treating physicians not involved in data collection or analysis. No therapeutic interventions were performed. As this was a non-interventional study, a clinical trial number was not required. The study adhered to the Declaration of Helsinki.

Powered by Editorial Manager® and ProduXion Manager® from Aries Systems Corporation

**Format for specific study types**<br><br>**Human Subject Research (involving human participants and/or tissue)**<br>• Give the name of the institutional review board or ethics committee that approved the study<br>• Include the approval number and/or a statement indicating approval of this research<br>• Indicate the form of consent obtained (written/oral) or the reason that consent was not obtained (e.g. the data were analyzed anonymously)<br><br>**Animal Research (involving vertebrate animals, embryos or tissues)**<br>• Provide the name of the Institutional Animal Care and Use Committee (IACUC) or other relevant ethics board that reviewed the study protocol, and indicate whether they approved this research or granted a formal waiver of ethical approval<br>• Include an approval number if one was obtained<br>• If the study involved *non-human primates*, add *additional details* about animal welfare and steps taken to ameliorate suffering<br>• If anesthesia, euthanasia, or any kind of animal sacrifice is part of the study, include briefly which substances and/or methods were applied<br><br>**Field Research**<br><br>Include the following details if this study involves the collection of plant, animal, or other materials from a natural setting:<br>• Field permit number<br>• Name of the institution or relevant body that granted permission	
**Data Availability**<br><br>Authors are required to make all data underlying the findings described fully available, without restriction, and from the time of publication. PLOS allows rare exceptions to address legal and ethical concerns. See the PLOS Data Policy and FAQ for detailed information.	Yes - all data are fully available without restriction

A Data Availability Statement describing where the data can be found is required at submission. Your answers to this question constitute the Data Availability Statement and **will be published in the article**, if accepted.<br><br>**Important:** Stating 'data available on request from the author' is not sufficient. If your data are only available upon request, select 'No' for the first question and explain your exceptional situation in the text box.<br><br>Do the authors confirm that all data underlying the findings described in their manuscript are fully available without restriction?	
**Describe where the data may be found in full sentences. If you are copying our sample text, replace any instances of XXX with the appropriate details.**<br><br>• If the data are **held or will be held in a public repository**, include URLs, accession numbers or DOIs. If this information will only be available after acceptance, indicate this by ticking the box below. For example: *All XXX files are available from the XXX database (accession number(s) XXX, XXX.).*<br>• If the data are all contained **within the manuscript and/or Supporting Information files**, enter the following: *All relevant data are within the manuscript and its Supporting Information files.*<br>• If neither of these applies but you are able to provide **details of access elsewhere**, with or without limitations, please do so. For example:<br><br>*Data cannot be shared publicly because of [XXX]. Data are available from the XXX Institutional Data Access / Ethics Committee (contact via XXX) for researchers who meet the criteria for access to confidential data.*<br><br>*The data underlying the results presented in the study are available from (include the name of the third party*	All relevant data are within the manuscript and its Supporting Information files.

and contact information or URL).<br>• This text is appropriate if the data are owned by a third party and authors do not have permission to share the data.<br><br>* typeset	
Additional data availability information:	

Powered by Editorial Manager® and ProduXion Manager® from Aries Systems Corporation

Manuscript Click here to access/download;Manuscript;renamed_4e18f.docx

Incidence and Risk Factors of Calcium Channel Blocker–Related Edema in Hypertensive Patients: A Multicenter Retrospective Cohort Study

Koricho Simie Tolla[1*], Gashaw Solela[2¥], Getachew Wondafrash[3], Abay Burusie[4], Gebi Agero[4], Dureti Desta Garoma[4], Wubshet Abraham Alemu[1], Bereket Sinshaw Engida[5], Surafel Mekasha Woldeyes[4], Berhanu Moges Abera[2], Mulualem Alemayehu Gebreselassie[6]

[1]Department of Internal Medicine, College of Health Sciences, Arsi University, Asella, Ethiopia

[2]Divison of Cardiology, College of Health Sciences, Addis Ababa University, Addis Ababa, Ethiopia

[3]Division of Nephrology, Department of Internal Medicine, Yekatit 12 Hospital Medical College, Addis Ababa, Ethiopia

[4]Department of Public Health, College of Health Sciences, Arsi University, Asella, Ethiopia

[5]Yehuleshet Specialty Clinic, Addis Ababa, Ethiopia

[6]Division of Cardiology, ICMC General Hospital, Addis Ababa, Ethiopia

*ORCID ID: 0009-0006-1825-4502

[¥]Correspondence: Gashaw Solela, gashawsol@gmail.com, ORCID ID: 0000-0002-2233-927

Abstract

Background: Hypertension is a major risk factor for cardiovascular disease and remains the leading cause of mortality worldwide. Calcium channel blockers (CCBs) are commonly used to lower blood pressure because they are effective and affordable. However, CCBs can cause vasodilatory adverse effects, including peripheral edema, which may lead to additional therapy and affect adherence. This study assessed the incidence and risk factors of CCB-related edema among hypertensive patients in Ethiopia.

Methods: This retrospective multicenter cohort study involved interviews and reviews of medical records of adults (aged ≥18 years) with essential hypertension who were prescribed calcium channel blockers (CCBs) between July 15 and August 14, 2025. A total of 292 participants were selected using systematic random sampling. A structured questionnaire was used to collect sociodemographic and clinical data. Descriptive statistics summarized baseline characteristics. Time-to-event analysis with the log-rank test assessed the duration from CCB initiation to edema onset. Binary and multivariate logistic regression analyses identified factors associated with CCB-related edema, and a p-value <0.05 was considered statistically significant.

Results: Among 292 participants (mean age 58.2 years; 53.4% female), 20.9% had diabetes mellitus and 16.8% had dyslipidemia. Amlodipine was the most frequently prescribed CCB (94.8%). Peripheral edema developed in 38.7% of patients, with a mean onset time of 8.3 weeks. In multivariate analysis, only longer daily standing duration was significantly associated with edema (AOR = 1.92; 95% confidence interval: 1.03–3.58; p = 0.041). Time-to-event analysis showed a progressive increase in edema risk with continued CCB use. Patients receiving amlodipine 10 mg daily had a greater (42.5% vs. 33%) and earlier risk of edema than those on 5 mg amlodipine daily; log-rank p = 0.003).

Conclusions: Peripheral edema occurred in over one-third of hypertensive patients on CCBs, appearing on average after eight weeks. Longer daily standing and higher CCB doses increased risk and earlier onset. Clinicians should inform patients and avoid unnecessary interventions. Further research on upright posture and edema is warranted.

Keywords: Adverse drug reactions; amlodipine; antihypertensive therapy; calcium channel blockers; Ethiopia; hypertension; peripheral edema.

Introduction

Hypertension is defined as a persistent systolic blood pressure (SBP) of at least 130 mmHg or a diastolic blood pressure (DBP) of at least 80 mmHg and is associated with an increased risk of cardiovascular disease (CVD) events, including coronary heart disease, heart failure, stroke, and death [1]. The global prevalence of hypertension has doubled over the past three decades, with nearly two-thirds of affected individuals residing in low- and middle-income countries (LMICs) [2, 3]. Despite this high burden, treatment and control rates remain low in many regions, particularly in sub-Saharan Africa [2]. In Ethiopia, hypertension has become a growing public health problem, with urban prevalence among adults reported to be as high as 30.3% [4].

Randomized controlled trials (RCTs) have consistently demonstrated that antihypertensive therapy diminishes cardiovascular morbidity and mortality [5]. The Eighth Joint National Committee (JNC 8) guidelines recommend that initial therapy should include a thiazide diuretic, calcium channel blocker (CCB), angiotensin-converting enzyme (ACE) inhibitor, or angiotensin receptor blocker (ARB) [6]. Among these, CCBs and ARBs are the most frequently prescribed drug classes worldwide. Multiple studies have identified CCBs as the most commonly used antihypertensive medications [7]. Similarly, studies from Ethiopia have identified CCBs, along with ACE inhibitors and thiazide diuretics, as the most commonly prescribed antihypertensive agents [8, 9].

Dihydropyridine (DHP) CCBs are associated with vasodilatory adverse effects, including peripheral edema [10]. This edema is thought to result from increased capillary hydrostatic pressure due to greater dilation of pre-capillary arterioles compared to post-capillary venules [11]. Peripheral edema not only causes discomfort but may also lead to additional investigations, the prescription of loop diuretics, and poor adherence to therapy. Patients on DHP-CCBs are reported to be up to 60% more likely to receive loop diuretics than those on other antihypertensives [10, 12].

The reported incidence of CCB-induced peripheral edema varies widely. Meta-analyses of RCTs have shown a pooled incidence of around 10.7% [13], while some observational studies have reported rates as high as 29% [14, 15]. This variation may be due to differences in the design of the studies, the people who took part, or environmental or genetic factors. Several potential risk modifiers have been identified, including CCB type, dosage, duration of use, age, sex, comorbidities (such as diabetes, dyslipidemia, chronic obstructive pulmonary disease, cerebrovascular disease, and coronary artery disease), and upright posture [13, 16-18]. However, the influence of many of these factors remains inconsistent across studies.

Given the lack of local data and potential differences in patient characteristics, this study aimed to determine the incidence and identify risk factors of CCB-related peripheral edema among hypertensive patients treated at two teaching hospitals in Ethiopia. Findings from this study are expected to raise awareness among clinicians, improve patient counseling, and help reduce unnecessary investigations and additional drug therapy related to CCB-induced edema.

Materials and methods

Study design, area, and period

A multicenter retrospective cohort study was conducted by reviewing medical records and interviewing adult patients (aged ≥18 years) diagnosed with essential hypertension who were prescribed CCBs. The study was carried out from July 15 to August 14, 2025, at Asella Teaching and Referral Hospital (ATRH) and Yekatit 12 Hospital Medical College (Y12HMC). These hospitals were purposefully selected to capture diverse patient populations, with ATRH serving suburban and rural communities and Y12HMC serving an urban population. Both institutions have well-established hypertension follow-up clinics that provide continuous care, maintain comprehensive medical records, and are staffed by residents, internists, fellows, and subspecialists, ensuring reliable sources of clinical and patient information for this study.

Source and study population

The source population included all adult patients (aged ≥18 years) diagnosed with essential hypertension and attending follow-up clinics at ATRH and Y12HMC who had been prescribed CCBs as part of their treatment regimen. The study population comprised those patients who attended follow-up during the study period and had complete medical records. Patients were excluded if they were non-compliant with treatment or if data on CCB usage or peripheral edema were missing.

Eligibility criteria

Adult patients aged 18 years and above, diagnosed with essential hypertension and prescribed CCBs for at least six months, or who discontinued CCB therapy earlier due to the development of peripheral edema, were eligible for inclusion. All types of CCBs, including amlodipine and nifedipine, were considered. Patients with pre-existing conditions that could contribute to edema, such as heart failure, renal insufficiency, deep vein thrombosis, venous insufficiency, chronic liver disease, hypothyroidism, pregnancy, or other relevant conditions, were excluded. Additionally, patients with incomplete medical records regarding history, CCB use, or development of edema, as well as those unable to provide informed consent, were not included in the study.

Sample size determination and sampling technique

The sample size was calculated based on a Nigerian prospective cohort study reporting a 29% incidence of peripheral edema among patients prescribed amlodipine or nifedipine [15]. Using a single population proportion formula with 95% confidence and 80% power, the initial sample size was 316. Considering that high-dose CCBs increase the risk of edema 2.8-fold [13], Fleiss' formula with continuity correction yielded 318. With a total hypertensive patient population of approximately 2,500 at both centers, and 50% on CCB therapy, the source population was estimated at 1,250. Applying the finite population correction resulted in an adjusted sample size of 253. Accounting for a 10% non-response rate, the final sample size was 278.

A consecutive sampling technique was used, enrolling all eligible patients attending hypertension follow-up clinics during the study period until the target sample size was reached. Of the total sample, 52% (n = 145) were recruited from ATRH and 48% (n = 133) from Y12HMC. Systematic random sampling was used to select patients attending referral clinics during the study period. If the sampled patient was not a CCB user, the CCB user following him/her was selected as a sample.

Study variables

The dependent variable was the incidence of calcium channel blocker (CCB)-related peripheral edema. The independent variables included sociodemographic factors (age, gender, and occupation), duration of daily upright posture, comorbid conditions (diabetes, chronic obstructive pulmonary disease, cerebrovascular accidents, coronary artery disease, dyslipidemia, and others), body mass index, and medication history. Medication history encompassed the type and dosage of CCBs prescribed, duration of therapy before discontinuation or edema development, and the use of other antihypertensive medications, including ACE inhibitors, angiotensin receptor blockers, thiazides, and beta-blockers. Among these, the specific dosage of CCB was considered the primary independent variable.

Operational definitions

CCB user: An adult patient with essential hypertension who has used a CCB for at least six months or who discontinued CCB therapy due to the development of peripheral edema. The primary CCBs in this study were amlodipine and nifedipine.

Posture: The predominant body position maintained during daily activities, classified as upright, sitting, or recumbent/lying down.

Adherence to salt restriction: Consumption of ≤5 g of table salt per day (approximately one teaspoon), in line with World Health Organization recommendations.

Data collection procedure

Data were collected using a structured questionnaire administered through the Kobo Toolbox platform. Demographic information and selected details on edema detection and progression were obtained via patient interviews, while comorbidities, physical examination findings, and medication histories were extracted from medical records. Data collection was performed by a team of four internal medicine residents and two internists, under the supervision of an internist, a cardiology fellow, and a nephrologist. Despite differences in training levels, the assessment of edema and completion of the questionnaire were considered straightforward, minimizing potential inter-observer variability.

Data analysis

Data were checked for completeness and analyzed using SPSS version 27 (IBM Corporation, Armonk, NY, USA). Descriptive statistics, including frequency distributions and measures of central tendency and dispersion, were used to summarize demographic and clinical characteristics of the study population. Time-to-event (survival) analysis was performed to evaluate the interval from initiation of CCB therapy to the development of peripheral edema, stratified by CCB dose, with patients who did not develop edema during follow-up being censored. Binary logistic regression was used to estimate odds ratios for the occurrence of edema, while multivariable logistic regression adjusted for potential confounders and assessed the independent associations of multiple risk factors with the outcome. Statistical significance was defined as a p-value < 0.05.

Data quality assurance

A pretest was conducted on 5% of the medical records to evaluate the clarity, feasibility, and consistency of the questionnaire. Data were collected by trained physicians under the supervision of the principal investigators. The completeness, consistency, and accuracy of the data were regularly monitored, and any discrepancies were promptly addressed to minimize information bias and ensure data reliability.

Results

Sociodemographic data

A total of 292 patients met the inclusion criteria and were included in the analysis. The mean age was 58.2 years, and 53.4% were female. Mean age was similar between participants who did and did not develop peripheral edema (58.27 vs. 58.22 years). The mean duration of hypertension was 47.9 months, ranging from 1 to 360 months. Regarding occupation, 37% of participants were retired, 20.5% were farmers, 14% were unemployed, and 10.3% were merchants (Table 1). On average, participants reported spending 2.9 hours per day standing (standard deviation [SD] = 2.0; range = 0–10 hours). The mean body weight was 68.0 kg (SD = 11.8; range = 40–115 kg).

Comorbidities were common, with diabetes mellitus in 20.9%, dyslipidemia in 16.8%, stroke in 9.6%, ischemic heart disease in 8.6%, and neuropathy in 8.2% of patients. Chronic kidney disease, HIV infection, and other conditions were less frequent, while 49.7% of participants had no additional medical conditions (Table 1). At the time of the most recent visit or edema assessment, blood pressure was controlled in 64% of participants, with a mean systolic BP of 134.6 mmHg (range 80–207) and a mean diastolic BP of 78.9 mmHg (range 51–130).

Table 1 Socio-demographic characteristics and comorbidities of hypertensive patients taking CCB at ATRH and Y12HMC, Ethiopia, 2025 (N = 292)

Variable	Number	Percentage
Total patients (n)	292	100.0
Sex		
Female	156	53.4
Male	136	46.6
Occupation		
Retired	108	37.0
Farmer	60	20.5
Merchant/Trader	30	10.3
Office worker	21	7.2
Others	25	8.6
Comorbidities		
Diabetes mellitus	61	20.9
Dyslipidemia	49	16.8
Stroke	28	9.6
Ischemic heart disease	25	8.6
Neuropathy	24	8.2
HIV Infection	9	3.1
Chronic kidney disease	2	0.7
Psychiatric condition	2	0.7

Medication history and adverse events

The most commonly prescribed CCB was amlodipine (95%), followed by nifedipine (4.5%). High-dose CCB was administered to 57.2% of patients (Fig. 1). Concomitant antihypertensive therapy was used in 57.2% of participants, with ACE inhibitors prescribed to 49.1% and thiazide diuretics to 26.9% of these patients (Table 2).

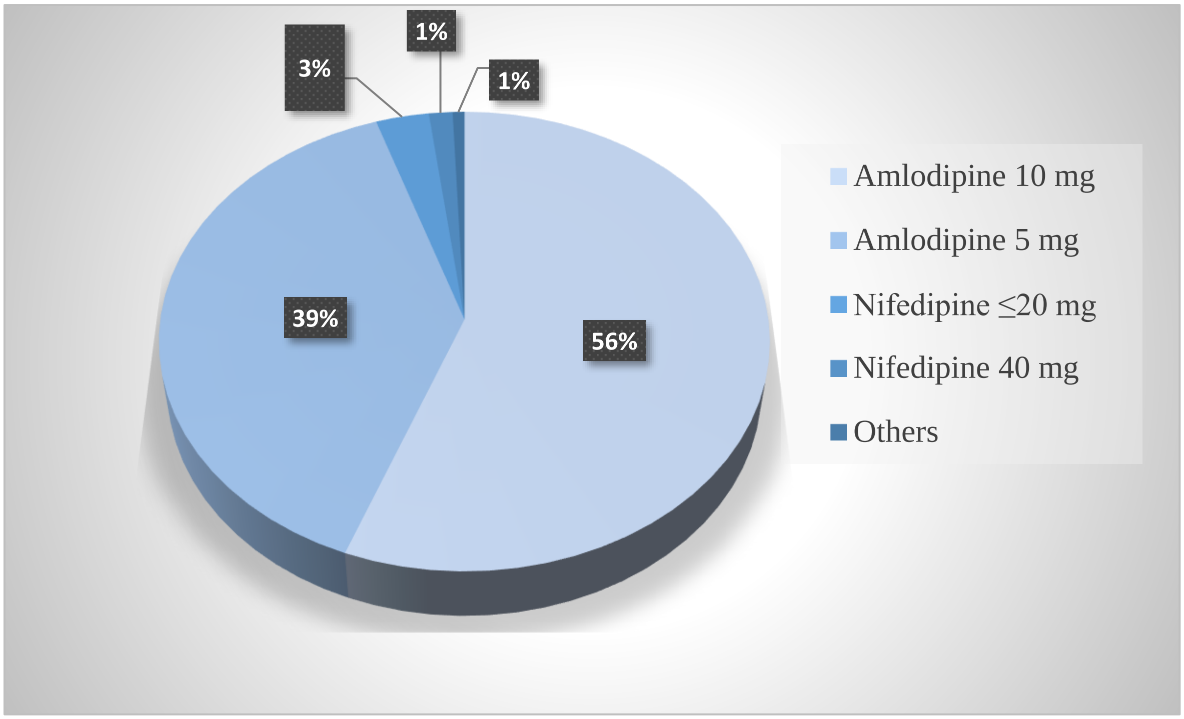

Fig. 1 Pie chart for the types and daily doses of CCBs used among hypertensive patients at ATRH and Y12HMC, Ethiopia, 2025 (N = 292)

Table 2 Additional antihypertensives used among hypertensive patients taking CCB at ATRH and Y12HMC, Ethiopia, 2025 (N = 292)

Concomitant antihypertensive drugs	Frequency	Percentage
ACEi	82	49.1
Thiazide diuretics	45	26.9
ACEi + thiazide diuretics	20	12
ARBs	6	3.6
ARBs + thiazide diuretics	5	3
ACEi + BB	3	1.8
ACEi + BB + others	2	1.2
Other combinations	4	2.4
Total	167	100.0

Abbreviations: ACEi, angiotensin-converting enzyme inhibitor; ARB, angiotensin receptor blocker; BB, beta-blocker

During follow-up, 38.7% of patients developed peripheral edema, with a mean onset of 8.3 weeks (range: 1–55 weeks) after initiating CCB therapy. Edema was predominantly bilateral and occurred more often in patients on high-dose therapy compared to low-dose therapy (42.5% vs. 33%). Patients self-reported edema in 84.9% of cases. Other commonly reported adverse effects included headache (14%), constipation (11.3%), and dizziness or lightheadedness (9.6%).

Risk factors

Binary logistic regression showed that longer daily standing duration and high-dose CCB therapy were associated with edema development. In multivariate analysis, only daily standing duration remained significant (AOR: 1.92; 95% CI: 1.03–3.58; P = 0.041), while high-dose CCB approached significance (AOR: 1.58; 95% CI: 0.97–2.57; P = 0.068). No comorbid conditions or other adverse effects, including headache and constipation, were associated with edema (Table 3).

Table 3 Logistic regression on risk factors for edema among hypertensive patients taking CCB at ATRH and Y12HMC, Ethiopia, 2025 (N = 292)

Variable	COR (95% CI)	p-value	AOR (95% CI)	p-value
Age	1.01 (0.99-1.03)	0.48	-	-
Sex (male)	1.60 (0.92-2.80)	0.099	-	-
Duration of daily standing	1.2(1.02-1.3)	0.02	1.92(1.026-3.58)	0.041
High-dose CCB	1.45 (1.018-2.06)	0.039	1.58(0.97-2.57)	0.068
Use of other antihypertensive medications	0.60 (0.36-1.02)	0.06	-	-
Controlled BP	0.72 (0.44-1.17)	0.18	-	-
Other CCB side effects	1.93 (0.67-5.56)	0.23	-	-
Physical exercise	1.66 (0.55-4.99)	0.37	-	-
Adherence to salt restriction	1.37 (1.56-3.35)	0.49	-	-
Current smoking status	0.67 (.062-7.18)	0.74	-	-
Daily alcohol consumption	4.91 (0.74-32.66)	0.10	-	-

Abbreviations: BP, blood pressure; CCB, calcium channel blocker

Management of CCB-related edema

Following edema onset, management strategies varied. Drug adjustment, including discontinuation, was performed in 58.4% of patients, and additional investigations were conducted in 23.9%. Diuretics were added in 22.3% of cases, whereas no patients received ACE inhibitors or ARBs. A minority of patients (17.7%) continued therapy with conservative measures, such as lifestyle modifications.

Time-to-event analysis

Time-to-event analysis, stratified by daily CCB dose (high vs. low), showed a progressive increase in the risk of edema with longer duration of CCB use. Participants receiving amlodipine 10 mg experienced a higher and earlier risk of edema compared to those taking 5 mg (Fig. 2). The difference between the groups was statistically significant by the log-rank test (P = 0.003; Log Rank [Mantel-Cox] = 15.703). Analysis was restricted to participants taking amlodipine, which accounted for nearly 95% of the study population.

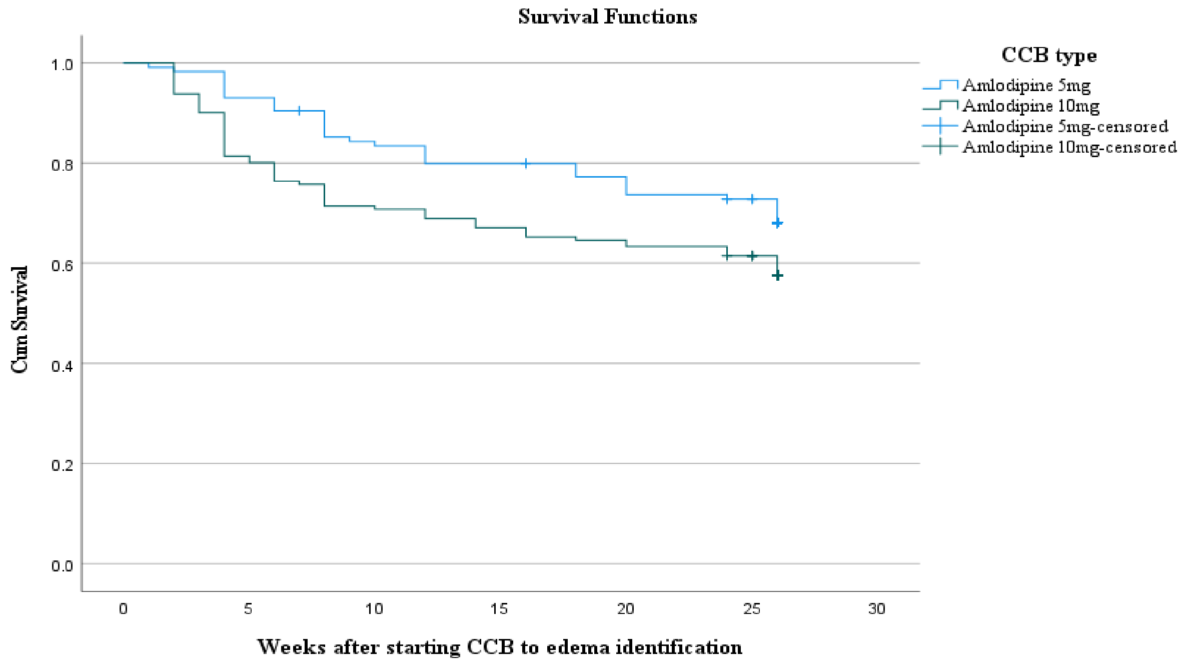

Fig. 2 Kaplan-Meier curve for development of edema among hypertensive patients taking CCB at ATRH and Y12HMC, Ethiopia, 2025 (N = 292)

Discussion

In this study, we retrospectively examined the incidence of CCB-related edema among 292 patients with hypertension at two hospitals in Ethiopia. The mean age of 58.2 years was comparable to that reported in one of the large meta-analyses by Makani et al. [13]. Based on the results of this study, the incidence of CCB edema was more common among the Ethiopian population than reports from other locations. Incidence rates from studies and meta-analyses ranged from 10.7% to 29%, which are lower compared to our finding of 38.7% [13-15].

The average time to identify edema in our study was 8.3 weeks (58.1 days), which is closer to the Indian study of 69 days [14] compared to a much shorter duration, such as 27.3 days, as reported by a different study [20]. This difference may be explained by the delayed reporting by patients, who were responsible for identifying edema in 85% of cases. This is supported by the fact that edema was reported as early as 1 week after initiating CCB in this study. This suggests that active monitoring for edema by physicians after initiating CCBs could lead to earlier detection.

In agreement with several other studies, this study also showed significantly higher incidence of edema in patients using higher doses of CCB, particularly in participants taking amlodipine 10 mg compared to 5 mg [13]. There is also data that supports a progressive increase in the incidence of edema with longer duration of CCB use, which is the case in our study, as illustrated by the Kaplan-Meier curve in the results section [15]. Not just higher incidence, this curve also showed earlier onset of edema among high-dose CCB users. This is supported by the biological plausibility of high-dose CCB resulting in a higher effect on precapillary vessels and subsequently increasing capillary hydrostatic pressure and edema.

Several modifiers of CCB-related edema have been well reported by studies, including upright posture, warmth, older age, and female gender [21]. Our study showed a significant association with the duration of upright posture. This is due to the upright posture providing an additional gravitational contribution to increased hydrostatic pressure and the development of edema [16, 17]. One study showed an association of CCB edema with comorbid conditions, including diabetes, COPD, coronary artery disease, and dyslipidemia, which our study didn’t evidence. This is likely due to the fact that, except for diabetes, other comorbid conditions were less frequent in our study.

The use of RAS inhibitors, such as ACE inhibitors, has been shown to reduce the incidence and severity of CCB-related edema [13, 16, 17]. Although there was a tendency to decrease the risk of CCB edema with concomitant use of ACEi and/or thiazides with an odds ratio of 0.60 (95% 0.36-1.02, $p = 0.06$), it didn't reach statistical significance. This may be due to the low number of participants using these medications.

Lack of acknowledgment of edema as an adverse effect of CCBs by clinicians might result in inadvertent use of additional interventions. One of the adverse effects of CCB-related edema is the sequential use of diuretics, which may expose patients to drug-related side effects like acute kidney injury. A large cohort study reported that CCB users experienced 60% higher rates of being subsequently dispensed a loop diuretic compared to other antihypertensive drug users [12]. Although we cannot directly compare this figure, our study found that 22.3% of participants were prescribed to use diuretics. Additionally, nearly 24% of our patients underwent additional laboratory and imaging tests after the detection of edema.

Conclusion

CCB-related edema is common in Ethiopian patients with hypertension and is associated with longer daily standing and higher doses. Many patients undergo unnecessary tests or receive diuretics for this side effect. Clinicians should recognize its high incidence, inform patients, and avoid unnecessary interventions. Further research on the role of daily upright posture in edema development is warranted.

Ethics approval and consent to participate

Ethical clearance was obtained from the College of Health Science, Arsi University, Ethical Review Committee (Ref. No: A/U/H/S/C/120/13619/17). Written informed consent was obtained from all participants. Data were anonymized to protect confidentiality, and only relevant research information was collected. Clinical care and research activities were strictly separated, with treating physicians not involved in data collection or analysis. No therapeutic interventions were performed. As this was a non-interventional study, a clinical trial number was not required. The study adhered to the Declaration of Helsinki.

Consent for publication

Not applicable.

Data availability statement

The datasets used or analyzed during the current study are available from the corresponding author upon reasonable request.

Competing interests

The authors declare that they have no competing interests.

Funding

No funding was obtained.

Author contributions

KST was involved in conceptualization, supervision, methodology, formal analysis, and writing – original draft. GS, GW, AB, and GA were involved in supervision, methodology, formal analysis, and writing – review and editing. DDG, WAA, BSE, SMW, BMA, and MAG were involved in formal analysis, validation, and writing – review and editing. All authors reviewed and approved the final manuscript.

Acknowledgments

The authors extend their gratitude to the study participants and the healthcare personnel at ATRH and Y12HMC referral clinics for their valuable support.

References

1. Carey RM, Moran AE, Whelton PK. Treatment of Hypertension: A Review. JAMA. 2022 Nov 8;328(18):1849–61.

2. Zhou B, Carrillo-Larco RM, Danaei G, Riley LM, Paciorek CJ, Stevens GA, et al. Worldwide trends in hypertension prevalence and progress in treatment and control from 1990 to 2019: a pooled analysis of 1201 population-representative studies with 104 million participants. The Lancet. 2021 Sept 11;398(10304):957–80.

3. Hypertension [Internet]. [cited 2025 Jan 18]. Available from: https://www.who.int/news-room/fact-sheets/detail/hypertension

4. Legese N, Tadiwos Y. <p>Epidemiology of Hypertension in Ethiopia: A Systematic Review</p>. IBPC. 2020 Oct 15;13:135–43.

5. James PA, Oparil S, Carter BL, Cushman WC, Dennison-Himmelfarb C, Handler J, et al. 2014 Evidence-Based Guideline for the Management of High Blood Pressure in Adults: Report From the Panel Members Appointed to the Eighth Joint National Committee (JNC 8). JAMA. 2014 Feb 5;311(5):507–20.

6. Armstrong C. JNC 8 Guidelines for the Management of Hypertension in Adults. afp. 2014 Oct 1;90(7):503–4.

7. Jarari N, Rao N, Peela JR, Ellafi KA, Shakila S, Said AR, et al. A review on prescribing patterns of antihypertensive drugs. Clin Hypertens. 2016 Mar 27;22(1):7.

8. Yazie TS, Yimer YS, Belete AM, Desta GT. Prescribing pattern of anti-hypertensive medications among hypertensive outpatients at selected hospitals of South Gondar Zone, Amhara, Ethiopia: a hospital based cross sectional study. BMC Pharmacol Toxicol. 2022 Dec 30;23(1):97.

9. Abegaz TM, Tefera YG, Abebe TB. Antihypertensive drug prescription patterns and their impact on outcome of blood pressure in Ethiopia: a hospital-based cross-sectional study. Integrated Pharmacy Research and Practice. 2017 Jan 27;6:29–35.

10. Chrysant SG. Proactive Compared With Passive Adverse Event Recognition: Calcium Channel Blocker–Associated Edema. The Journal of Clinical Hypertension. 2008;10(9):716–22.

11. de la Sierra A. Mitigation of calcium channel blocker-related oedema in hypertension by antagonists of the renin–angiotensin system. J Hum Hypertens. 2009 Aug;23(8):503–11.

12. Savage RD, Visentin JD, Bronskill SE, Wang X, Gruneir A, Giannakeas V, et al. Evaluation of a Common Prescribing Cascade of Calcium Channel Blockers and Diuretics in Older Adults With Hypertension. JAMA Internal Medicine. 2020 May 1;180(5):643–51.

13. Makani H, Bangalore S, Romero J, Htyte N, Berrios RS, Makwana H, et al. Peripheral edema associated with calcium channel blockers: incidence and withdrawal rate--a meta-analysis of randomized trials. J Hypertens. 2011 July;29(7):1270–80.

14. Dalal J, Sawhney JP, Jayagopal PB, Hazra PK, Khan MY, Gaurav K, et al. A Retrospective, Observational, EMR-Based Real-World Evidence Study to Assess the Incidence of Pedal Edema in Essential Hypertensive Patients on Amlodipine or Cilnidipine. International Journal of Hypertension. 2022;2022(1):6868143.

15. Otor Onah P, Idoko C, Abdulateef S. Prevalence of Calcium Channel Blocker Induced Ankle Edema and its Treatment in a Cohort of Newly Diagnosed Hypertensive Patients. International Journal of Pharmaceutical Sciences and Medicine. 2022 May 30;7:97–108.

16. Sica DA. Calcium channel blocker-related periperal edema: can it be resolved? J Clin Hypertens (Greenwich). 2003;5(4):291–4, 297.

17. Galappatthy P, Waniganayake YC, Sabeer MIM, Wijethunga TJ, Galappatthy GKS, Ekanayaka RA. Leg edema with (S)-amlodipine vs conventional amlodipine given in triple therapy for hypertension: a randomized double blind controlled clinical trial. BMC Cardiovascular Disorders. 2016 Sept 1;16(1):168.

18. Khadka S, Joshi R, Shrestha DB, Shah D, Bhandari N, Maharjan M, et al. Amlodipine-Induced Pedal Edema and Its Relation to Other Variables in Patients at a Tertiary Level Hospital of Kathmandu, Nepal. Journal of Pharmacy Technology. 2019 Apr 1;35(2):51–5.

19. Sangam K, Devireddy P, Konuru V. CALCIUM CHANNEL BLOCKERS INDUCED PERIPHERAL EDEMA. 2016 June 1;7:290–3.

21. Liang L, Kung JY, Mitchelmore B, Cave A, Banh HL. Comparative peripheral edema for dihydropyridines calcium channel blockers treatment: A systematic review and network meta-analysis. J Clin Hypertens (Greenwich). 2022 May;24(5):536–54.

Click here to access/download
Supporting Information
Ethical approval letter.pdf
