# Supplementary material for: Incidence and risk factors of calcium channel blocker–related edema in hypertensive patients: A Multicenter retrospective cohort study
Source: PLoS One. 2026 Mar 19;21(3):e0336659. doi: 10.1371/journal.pone.0336659 (PMC13001965; doi:10.1371/journal.pone.0336659)
Supplement: Attachment — Submitted filename: PLOS One Review F Gueyffier V2.docx [file pone.0336659.s005.docx]

**Review of the manuscript “Incidence and Risk Factors of Calcium Channel Blocker–Related Edema in Hypertensive Patients: A Multicenter Retrospective Cohort Study” by Gashaw Solela et al.**

**Reviewer:** François GUEYFFIER

1. **The study presents the results of original research.**

Yes, results were specifically obtained following a protocol that was submitted to, and agreed by, a local ethical committee.

2. **Results reported have not been published elsewhere.**

No, as far as I can ascertain… Another publication is unlikely due to the delay.

3. **Experiments, statistics, and other analyses are performed to a high technical standard and are described in sufficient detail.**

Almost: The sample size is justified and has been reached, but its justification lacks some details. Indeed, there is an appropriate power (80%), but we do not know what is the hypothesis behind. Is this the precision of the confidence interval for the estimate of the incidence of edema? Or is this the possibility to identify risk factors, i.e. to contrast the incidence or edema between two groups presenting or not the risk factor?

4. **Conclusions are presented in an appropriate fashion and are supported by the data.**

In general yes, but I would suggest to the authors some changes.

4.1. Definition of hypertension

Such a definition is arbitrary. The risk of cardiovascular events rises continuously for all levels of blood pressure^1^, and the lowest level could be adopted as well as more conservative ones. The issue behind such choice is obviously the association of a definite hypertension with an indication for pharmacological treatment. As a consequence, the lowest level of the definition will be associated with the largest volume of prescription. Guidelines have progressively moved towards lower levels along decades, from 160 mmHg initially re. systolic blood pressure to 130 mmHg as adopted by the authors. It is well established that guidelines are produced by experts who are full of conflicts of interest with pharmaceutical industry. The lower levels of hypertension definition along time is a likely explanation for the increase of the prevalence of hypertension mentioned by the authors.

Another possibility would be to chose as a definition of hypertension the level of blood pressure that is associated with a certainty that pharmacological treatment is beneficial. Guidelines acknowledge that there is weaker evidence for a benefit in mild hypertension, i.e. between 140 and 160 mmHg of systolic blood pressure. We established that there is no evidence for a favourable risk to benefit ratio in mild hypertension^2^. As a consequence, it is highly likely that the studied population is overtreated, confronted to side effects of pharmacological treatment without any solid hope for a benefit. This fact must be acknowledged in the introduction and in the discussion.

4.2. Methods of an observational retrospective study.

The strengths of the study are notable. The design is defined around the main objective of estimating the incidence of edema. They use medical files of known quality from two big hospitals with appropriate medical staff, and a supervision of the data extraction. The sampling procedure seems appropriate. Some aspects of the results suggest that there is a strong reality behind them: the relationship between dosage and the rate of edema; the increase of this rate along time of exposure.

The specific limitations of the experimental design must be better acknowledged by the authors.

First, there is no control group to compare the incidence of edema, which could be attributable to the exposure to CCBs or to other factors as well. The authors could have merely chosen to complete their study in comparing edema incidence with hypertensive people never treated by CCBs. The higher rate of edema they observe could be related to the fact that they attribute to CCBs edema that would have occurred under placebo. Placebo control in randomized trial is the best way to attribute causally an event to the treatment exposure, which is not possible here. In a meta-analysis of RCTs on edema with CCBs, authors found that 29% of edema observed on CCBs were also observed on placebo^3^.

Second, retrospective analyses are exposed to specific biases that must be discussed.

4.3. The coherence of these results with the existing literature is convincing, e.g. the standing position as a risk factor of edema, or the protective effect of ACE inhibitors against edema occurrence, well illustrated elsewhere^4^.

4.4. The conclusion that physicians should avoid unnecessary interventions is a truism, and has nothing specific to this population, or this treatment. CCBs are among the best BP lowering drugs to reduce the risk of stroke^5^. So the risk of oedema has to be balanced against the benefit from this drugs relative of stroke. And the majority of patients are likely to have mild hypertension without any solid hope for benefit.

5. **The article is presented in an intelligible fashion and is written in standard English.**

Yes, at least for a French reader !!!

6. **The research meets all applicable standards for the ethics of experimentation and research integrity.**

Yes, with informed consent collected for retrospective use of data collected within medical files.

7. **The article adheres to appropriate reporting guidelines and community standards for data availability.**

Yes. Data are said to be fully available without restriction.

1.	MacMahon S, Peto R, Cutler J, et al. Blood pressure, stroke, and coronary heart disease. Part 1, Prolonged differences in blood pressure: prospective observational studies corrected for the regression dilution bias. *Lancet* 1990; **335**(8692): 765-74.

2.	Diao D, Wright JM, Cundiff DK, Gueyffier F. Pharmacotherapy for mild hypertension. *Cochrane Database Syst Rev* 2012; **2012**(8): Cd006742.

3.	Makani H, Bangalore S, Romero J, et al. Peripheral edema associated with calcium channel blockers: incidence and withdrawal rate--a meta-analysis of randomized trials. *J Hypertens* 2011; **29**(7): 1270-80.

4.	Liang L, Kung JY, Mitchelmore B, Cave A, Banh HL. Comparative peripheral edema for dihydropyridines calcium channel blockers treatment: A systematic review and network meta-analysis. *J Clin Hypertens (Greenwich)* 2022; **24**(5): 536-54.

5.	Turnbull F. Effects of different blood-pressure-lowering regimens on major cardiovascular events: results of prospectively-designed overviews of randomised trials. *Lancet* 2003; **362**(9395): 1527-35.
