# Supplementary material for: Incidence and risk factors of calcium channel blocker–related edema in hypertensive patients: A Multicenter retrospective cohort study
Source: PLoS One. 2026 Mar 19;21(3):e0336659. doi: 10.1371/journal.pone.0336659 (PMC13001965; doi:10.1371/journal.pone.0336659)
Supplement: Attachment — Submitted filename: Response to Reviewers.docx [file pone.0336659.s007.docx]

**Response to Reviewers’ Comments**

| **Journal: PLOS ONE** | |
| --- | --- |
| **Manuscript ID: PONE-D-25-58381** | |
| **Title: Incidence and Risk Factors of Calcium Channel Blocker–Related Edema in Hypertensive Patients: A Multicenter Retrospective Cohort Study** | |
| **Academic Editor Comments** | **Authors’ Responses** |
| Please ensure that your manuscript meets PLOS ONE's style requirements, including those for file naming. | Thank you for your comment. We have revised the manuscript and all associated files to fully comply with PLOS ONE’s style and file-naming requirements. |
| Please amend the manuscript submission data (via Edit Submission) to include author Mulualem Alemayehu Gebreselassie. | Thank you for the notification. We have amended the manuscript submission data (via Edit Submission) to include author Mulualem Alemayehu Gebreselassie. |
| Please amend your authorship list in your manuscript file to include author Mulualem Alemayehu Gebreselassie. | Thank you for pointing that out. We have updated the authors' list at the beginning of the manuscript and revised the section on authors' contributions as well. |
| Please provide an amended Funding Statement declaring this commercial affiliation, as well as a statement regarding the Role of Funders in your study. | Thank you for this request. We would like to note that the authors with commercial affiliations made purely scholastic contributions. The manuscript has been revised accordingly. |
| Please also provide an updated Competing Interests Statement declaring this commercial affiliation, along with any other relevant declarations relating to employment, consultancy, patents, products in development, or marketed products, etc. | Thank you for this request. We would like to note that there is no competing interest to be mentioned regarding the commercial companies mentioned. The manuscript has been revised accordingly. |
| Your ethics statement should only appear in the Methods section of your manuscript. | Thank you for the suggestion. We have revised the manuscript so that the Ethical Statement now appears at the beginning of the Materials and Methods section. |
| Please include captions for your Supporting Information files at the end of your manuscript, and update any in-text citations to match accordingly. | Thank you for this helpful comment. Captions for all Supporting Information files have been added at the end of the manuscript, and all in-text citations have been revised accordingly to ensure consistency. |
| Please correct the definition of hypertension to the more accepted 140/90 mmHg, realizing that this is an arbitrary number from guidelines. | Thank you for your comment. We have updated the definition of hypertension to the widely accepted threshold of 140/90 mmHg, acknowledging that this value is guideline-based and somewhat arbitrary. |
| An important statistic that is missing is the number of people who stopped the drug due to the edema | Thank you for your comment. That was an important part of the study at its start. However, since the study required a diagnosis of CCB-related edema to be made after physician evaluation, it was challenging to include these patients solely on their reports. To the investigators' best knowledge, the number of patients who reported discontinuation before the visit was low, as follow-up visits are monthly. |
| **Reviewer 1 Comments** | **Authors’ Responses** |
| I do suggest reporting adjusted ORs (after defining what adjustment was done, or what "AOR" means), rather than arbitrarily saying one AOR is "significant" but another is not. | Thank you for your valuable feedback. We have now defined AOR in the “Data Analysis” section as the odds ratio from multivariable logistic regression adjusted for potential confounders, ensuring all associations are interpreted based on proper adjustment rather than arbitrary significance. |
| How early can edema occur from CCBs - if one checks before drug initiation (Day 0) could edema be present by Day 1 or Day 2, or only by Day 7 (1 week)? | Thank you for your comment. No patients developed edema within the first week of CCB therapy. None of the patients experienced edema during the first week of CCB therapy. Overall, 38.7% developed peripheral edema, occurring at a mean of 10.5 ± 8.3 weeks after treatment initiation. We have highlighted these changes in the revised manuscript. |
| Does edema relate in any way to antihypertensive effect? For example, if there is no change in BP, can edema still occur (assuming patient is taking the drug)??? | Thank you for this insightful comment. We have analyzed the relevant data accordingly and have incorporated these results into the revised manuscript to clarify this distinction. |
| **Reviewer 2 Comments** | **Authors’ Responses** |
| We did not find a study flow chart of excluded observations. We strongly recommend using flow charts because they provide an overview of the study design and more information about attrition. If you included a study flowchart in your supplemental files, we apologize for missing it. Our tool is not able to screen separate supplemental files. | Thank you for your comment. We acknowledge the importance of a study flowchart to summarize participant inclusion and attrition. We have now provided a study flowchart in the revised manuscript. |
| Sentence about attrition: not detected. Please provide information about the drop-out of subjects, or loss of animals or samples. This could be done using a study flow chart, or described in the text. | Thank you for your comment. This is not applicable to our study, as it was a retrospective cohort study and all eligible patient records were available for analysis. Therefore, no drop-outs, loss of subjects, or missing data occurred. However, we have provided a study flowchart to illustrate the selection of participants and the study population. |
| Blinding not detected. Please specify whether blinding was used at various phases of the experiment (e.g., blinding of patients, caregivers, outcome assessments, data analysis). | Thank for your comment. Blinding was not applicable in our study. |
| An explicit section about the limitations of this study was not found. We encourage authors to include a paragraph in the discussion that addresses study limitations. Every study has limitations. Describing these limitations helps readers to understand and contextualize the research. | We appreciate your suggestion. A dedicated paragraph has been added at the end of the Discussion section to transparently address the limitations of our study. |
| **Reviewer 3 Comments** | **Authors’ Responses** |
| Is this the precision of the confidence interval for the estimate of the incidence of edema? Or is this the possibility to identify risk factors, i.e. to contrast the incidence or edema between two groups presenting or not the risk factor? | Thank you for your comment. While we did not report a confidence interval for the overall incidence of edema, the 95% confidence intervals were used to assess the precision of the associations between potential risk factors and edema. |
| Conclusions: I would suggest to the authors some changes. | Thank you for your feedback. We have made some adjustments to the conclusion. |
| The definition of hypertension is arbitrary. | Thank you for your feedback. We have updated the definition of hypertension to the widely accepted threshold of 140/90 mmHg, acknowledging that this value is guideline-based and somewhat arbitrary. |
| The specific limitations of the experimental design must be better acknowledged by the authors.   - First, there is no control group to compare the incidence of edema - Retrospective analyses are exposed to specific biases that must be discussed. | Thank you for your comment. We greatly appreciate this input and have now included a dedicated paragraph on the limitations of our study in the Discussion section of the revised manuscript. |
| The conclusion that “physicians should avoid unnecessary interventions” is a truism, and has nothing specific to this population, or this treatment.  CCBs are among the best BP lowering drugs to reduce the risk of stroke. So the risk of oedema has to be balanced against the benefit from these drugs relative of stroke. And the majority of patients are likely to have mild hypertension without any solid hope for benefit. | We found your comment to be valid, as the statement was somewhat vague. Accordingly, we have revised the wording in the Discussion section. |
